# Interplay Between *Toxoplasma gondii*, Autophagy, and Autophagy Proteins

**DOI:** 10.3389/fcimb.2019.00139

**Published:** 2019-05-01

**Authors:** Carlos S. Subauste

**Affiliations:** ^1^Division of Infectious Diseases and HIV Medicine, Department of Medicine, Case Western Reserve University, Cleveland, OH, United States; ^2^Department of Pathology, Case Western Reserve University, Cleveland, OH, United States

**Keywords:** autophagy, parasite, CD40, IFN-gamma, cell signaling

## Abstract

Survival of *Toxoplasma gondii* within host cells depends on its ability of reside in a vacuole that avoids lysosomal degradation and enables parasite replication. The interplay between immune-mediated responses that lead to either autophagy-driven lysosomal degradation or disruption of the vacuole and the strategies used by the parasite to avoid these responses are major determinants of the outcome of infection. This article provides an overview of this interplay with an emphasis on autophagy.

Lysosomal degradation is an important mechanism of defense against numerous pathogens. This can be accomplished not only through the endocytic pathway but also through macroautophagy (called herein autophagy) (Levine et al., 2011). Autophagy is a homeostatic mechanism whereby large portions of cytosol or entire organelles are encircled by a double membrane (isolation membrane) leading to the formation of an autophagosome (Klionsky and Emr, 2000; Yoshimori, 2004; Mizushima et al., 2010). This structure fuses with lysosomes resulting in the formation of an autolysosome and cargo degradation (Mizushima et al., 2010).

Autophagy is dependent on a cascade of autophagy proteins (ATG). However, these proteins can have functions independent of autophagosome formation and lysosomal degradation (Subramani and Malhotra, 2013). This led to the use of the terms canonical and non-canonical autophagy where the latter was frequently used for processes that are non-degradative and/or not dependent on a core component(s) of the autophagy cascade [ATG3, ATG5, ATG7, Unc-51-like kinase 1 (ULK1), Beclin 1, and/or Phosphatidylinositol 3-kinase catalytic subunit type 3, PI3KC3, also known as VPS34] (Galluzzi et al., 2017). To avoid confusion, an expert panel recommended against the use of the terms “canonical”/“non-canonical,” and advised that the term autophagy be used solely for processes dependent on autophagosomes where cytosolic material (either endogenous or exogenous) is directed to a process that culminates with and is strictly dependent on lysosomal degradation (Galluzzi et al., 2017). The processes can be further characterized by stating the autophagy proteins they are dependent on Galluzzi et al. (2017). In this review, we summarize studies on the interplay between *T. gondii* and host autophagy as well as non-degradative processes controlled by autophagy proteins.

## Invasion of Host Cells by *T. gondii*

Tachyzoites of *T. gondii* infect virtually any nucleated cell and survive by residing in a compartment called the parasitophorous vacuole (PV). This vacuole is formed during active invasion of host cells, a process dependent on the parasite actin-myosin motor and sequential secretion of proteins from micronemes and rhoptries (Bradley and Sibley, 2007; Besteiro et al., 2011; Santos and Soldati-Favre, 2011). Once secreted from micronemes, *T. gondii* micronemal proteins (MICs) are expressed on the parasite surface and function as adhesins that interact with host cell membrane receptors (Carruthers and Tomley, 2008). MICs are expressed as multiprotein complexes that include MIC1/4/6, MIC3/8, MIC2/M2AP, and a complex of the microneme protein TgAMA1 with rhoptry neck proteins (Cerede et al., 2005; Huynh and Carruthers, 2006; Sheiner et al., 2010). MICs contain domains such as type I thrombospondin repeats, apple domains, epidermal growth factor (EGF) repeats, and integrin A domains (Tomley and Soldati, 2001; Anantharaman et al., 2007). The connection between transmembrane MICs and the parasite actin-myosin motor together with binding to host cell receptors enables the parasite to penetrate host cells (Soldati-Favre, 2008; Sibley, 2011). Following the release of MICs, rhoptries secrete a complex of neck proteins (RONs) containing RON2 that associates with the host cell membrane, plus RON4, RON5, and RON8 that are exposed to the host cell cytoplasm (Bradley and Sibley, 2007; Besteiro et al., 2011; Santos and Soldati-Favre, 2011). The complex forms a structure called moving junction that anchors the parasite to the host cell cytoskeleton during invasion (Bradley and Sibley, 2007; Besteiro et al., 2011; Santos and Soldati-Favre, 2011). Tachyzoites penetrate the host cell through the moving junction causing invagination of the host cell membrane. The moving junction also appears to function as a sieve that excludes host type I transmembrane proteins from entering the membrane that encircles the parasite as it penetrates the host cell (Mordue et al., 1999; Besteiro et al., 2011). Once invasion is completed, *T. gondii* resides within the PV. While host endocytic structures are delivered intact into the vacuolar space, there is no release of endosomal contents into the vacuole (Coppens et al., 2006). The lack of fusion with the endocytic compartment would be explained by the absence of host type I transmembrane proteins in the PV membrane (PVM) (Mordue et al., 1999; Besteiro et al., 2011).

## Autophagy Overview

Formation of the isolation membrane is dependent on recruitment of ATG proteins (Mizushima et al., 2010). Activation of both ULK1 and the complex that contains Beclin 1 and PI3KC3 drive the recruitment of ATG proteins to the isolation membrane promoting autophagosome formation and maturation (Chan et al., 2009; Itakura and Mizushima, 2010; Mizushima et al., 2010; Russell et al., 2013). ULK1 is the upstream kinase that triggers autophagy (Itakura and Mizushima, 2010). ULK1 is regulated by AMP-activated protein kinase (AMPK) and mechanistic target of rapamycin complex 1 (mTORC1), kinases that sense nutrient and energy status. In response to falling energy status, AMPK activates ULK1 and autophagy is stimulated (Chang et al., 2009; Egan et al., 2011; Kim et al., 2011; Mack et al., 2012). In contrast, ULK1 is inhibited by mTORC1 under nutrient rich conditions, leading to inhibition of autophagy (Chang et al., 2009). ULK1 undergoes membrane translocation upon activation by AMPK (Chang et al., 2009; Egan et al., 2011; Kim et al., 2011; Mack et al., 2012). Autophagosome biogenesis begins with the formation and activation of a ULK1-containing protein complex on membranes that express ATG9 (Papinski et al., 2014). ULK1 activates and recruits the Beclin 1–PI3KC3 complex to the membrane (Itakura and Mizushima, 2010). PI3KC3 causes production of PI3P at the membrane (Liang et al., 1999) and recruitment of PI3P-binding proteins that would act as scaffold for proteins that mediate membrane remodeling (Nascimbeni et al., 2017). Active Beclin-PI3KC3 triggers recruitment of ATG proteins that in turn function as two ubiquitin-like conjugation systems. In one cascade, ATG7 and ATG10 promote the conjugation of ATG5 to ATG12 (Mizushima et al., 1998). In the other cascade, ATG3 and ATG7 together with the ATG12-ATG5-ATG16L1 complex allow lipidation (phosphatidylethanolamide conjugation) of LC3 (ATG8) (Mizushima et al., 1998). Lipidated LC3 (LC3-II) is recruited to the autophagosome membrane (Kabeya et al., 2000) and allows substrate uptake by binding to several autophagy receptors (Stolz et al., 2014; Wild et al., 2014). Once the cargo is sequestered by the autophagosomes and through the effect of proteins that include Rab7 (Gutierrez et al., 2004), these structures fuse with lysosomes leading to the formation of an autolysosome.

Beclin 1 is regulated through protein-protein interactions. Beclin 1 binds proteins that promote autophagy (e.g., ATG14L) whereas binding to other proteins (e.g., Bcl-2 family members) inhibits autophagy (Pattingre et al., 2005; Sun et al., 2008; Matsunaga et al., 2009). Under basal conditions Bcl-2 binds to the BH3 domain of Beclin 1 preventing the association of Beclin 1 to PI3KC3 and the initiation of autophagy (Pattingre et al., 2005). Starvation stimulates autophagy in part because it triggers JNK1-dependent phosphorylation of Bcl-2 that releases Beclin 1 from Bcl-2 (Wei et al., 2008).

Autophagy proteins can be involved in cellular processes activated during intracellular infections that do not represent *bona fide* autophagy. LC3-associated phagocytosis (LAP) represents an example of such a process. LAP consists in the recruitment of LC3 and some other components of the autophagy pathway to single-membrane phagosomes that contain pathogens or dead cells that have been actively phagocytosed (Sanjuan et al., 2007). While LAP requires proteins that include ATG3, ATG5, ATG7, ATG12, ATG16L, Beclin 1, and PI3KC3, other proteins notably ULK1 are not involved in this process (Martinez et al., 2015). LAP is believed to result in faster fusion with lysosomes and plays a protective role against various pathogens (Sanjuan et al., 2007; Martinez et al., 2015). Autophagy proteins can be involved in additional mechanisms of anti-microbial activity that occur independently of the formation of autophagosomes and are not mediated by lysosomal degradation of the pathogen. In this regard, IFN-γ induces autophagy protein-dependent recruitment of GTPases that disrupt the integrity of the PV membrane (see below).

## Autophagy During *T. gondii* Infection

### CD40 Stimulates Autophagy and Triggers Autophagic Targeting of *T. gondii*

Autophagy can be stimulated by innate and adaptive immune mechanisms to degrade various pathogens (Levine et al., 2011). Pattern recognition receptors including TLR and NOD2 as well as cytokines such IFN-γ and type I interferon can stimulate autophagy (Shi and Kehrl, 2010; Gade et al., 2012; Matsuzawa et al., 2012; Chauhan et al., 2015). CD40 is a stimulator of autophagy that confers resistance against cerebral and ocular toxoplasmosis. CD40 is a member of the TNF receptor superfamily that is expressed on antigen presenting cells and various non-hematopoietic cells (Van Kooten and Banchereau, 2000). CD40 ligand (CD154) is expressed primarily on activated CD4^+^ T cells but is also present in activated platelets and plasma (Van Kooten and Banchereau, 2000). Studies in patients with congenital lack of functional CD154 (X-linked Hyper IgM syndrome) uncovered the central role of the CD40-CD154 pathway in protection against toxoplasmosis (Subauste et al., 1999). While the CD40-CD154 pathway promotes Th1-type cytokine response against *T. gondii* (Subauste et al., 1999; Reichmann et al., 2000), toxoplasmacidal activity induced by CD40 ligation in infected cells likely contributes to protection against the parasite (Reichmann et al., 2000; Portillo et al., 2010). Using peritoneal cells from *T. gondii*-infected mice, it has been proposed that CD40 plays a secondary role in parasite elimination in macrophages, although the CD4^+^ T cell–macrophage ratio (extent of CD40-CD154 interaction *in vitro*) and whether macrophages infected *in vitro* had undergone prior CD40-CD154 signaling *in vivo* were unclear (Zhao et al., 2007). Other studies demonstrated that CD154^+^ *T. gondii*-reactive CD4^+^ T cells induce anti-*T. gondii* activity in macrophages even if CD40 ligation occurs in cells already infected with *T. gondii* (Andrade et al., 2006). Importantly, studies in CD154^−/−^ and CD40^−/−^ mice established that this pathway is central for restricting parasite load in the brain and retina, and protecting against cerebral and ocular toxoplasmosis (Reichmann et al., 2000; Portillo et al., 2010), the two main forms of disease in humans.

Several lines of evidence indicate that CD40 stimulates autophagy and induces killing of *T. gondii* through autophagic targeting of the parasite, a phenomenon that occurs in hematopoietic and non-hematopoietic cells from both human and mice. CD40 ligation increases conversion of the autophagy protein LC3-I to LC3-II, as well as increases formation of autophagosomes and autolysosomes (autophagy flux). These events are dependent on ULK1, ATG5, ATG7, and Beclin 1 (Andrade et al., 2006; Portillo et al., 2010; Ogolla et al., 2013; Van Grol et al., 2013; Liu et al., 2015). In cells infected with *T. gondii*, CD40 ligation induces accumulation of mannose 6 phosphate receptor, Rab7, LAMP-1, LAMP-2, CD63, and cathepsin D around the PV as well as co-localization of these vacuoles with the acidotropic dye Lysotracker (Andrade et al., 2006; Portillo et al., 2010; Ogolla et al., 2013; Van Grol et al., 2013). Accumulation of lysosomal markers occurs around vacuoles that contain proteins secreted by parasite dense granules within the vacuolar lumen (Andrade et al., 2006). This indicates that the events triggered by CD40 represent fusion of the PV with lysosomes rather than being a consequence of phagocytosis since secretion of dense granule contents takes place during formation of PV but not during phagocytosis of *T. gondii*. Moreover, vacuole-lysosomal fusion (VLF) still occurs even if CD40 is engaged 18 h after infection (Andrade et al., 2006). VLF is preceded by accumulation of LC3 around the PV. Autophagy mediates VLF and killing of *T. gondii* since knockdown of ULK1, Beclin 1, PI3KC3, ATG5, or ATG7, expression of dominant negative Rab7 or pharmacologic inhibition of vacuolar ATPase or PI3K, and importantly incubation with lysosomal enzyme inhibitors ablate killing of *T. gondii* induced by CD40 (Andrade et al., 2006; Portillo et al., 2010; Van Grol et al., 2013). CD40 triggers VLF via autophagy rather than LAP since ULK1 is required for autophagy while LAP takes place independently of ULK1 (Martinez et al., 2015). In addition, the events triggered by CD40 ligation do not represent phagocytosis of the parasite.

**Figure 1:**
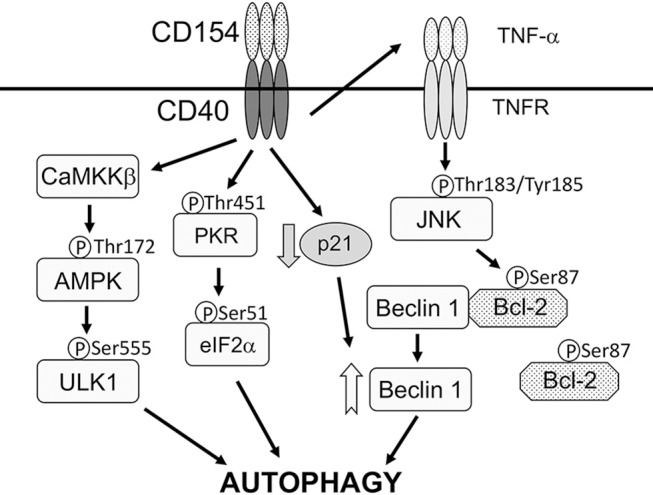
CD40 activates signaling pathways that stimulate autophagy. CD40 stimulates autophagy via four mechanisms. (1), CD40 induces CaMKKβ-mediated Threonine-172 AMPK phosphorylation that in turn causes Serine-555 ULK1 phosphorylation and ULK1-mediated autophagy. (2), CD40 induces autocrine secretion of TNF-α that causes JNK1/2-dependent phosphorylation of Bcl-2 at Serine 87 and dissociation of Bcl-2 from Beclin 1. (3), CD40 upregulates Beclin 1 protein levels likely through downregulation of p21. (4), CD40 activates PKR and eIF2α. CD40 may activate additional mechanisms that act on ULK1 and Beclin 1. CD40 causes autophagic killing of *T. gondii* that is dependent on ULK1, Beclin 1, PI3KC3, ATG5, ATG7, and lysosomal enzymes. Modified with permission from Liu et al. (2016).

CD40 stimulates autophagy via four mechanisms (Figure 1). First, CD40 induces CaMKKβ-mediated Threonine-172 AMPK phosphorylation, a marker of AMPK activation (Liu et al., 2016). In turn, AMPK signaling causes Serine-555 ULK1 phosphorylation and ULK1-mediated autophagy (Liu et al., 2016). Second, CD40 induces autocrine secretion of TNF-α that causes JNK1/2-dependent phosphorylation of Bcl-2 at Serine 87 and dissociation of Bcl-2 from Beclin 1 (Subauste et al., 2007; Liu et al., 2016). The latter process is known to allow binding of Beclin 1 to PI3KC3 and initiation of autophagy (Pattingre et al., 2005). Third, CD40 upregulates Beclin 1 protein levels *in vitro* and *in vivo* (Portillo et al., 2010). This effect appears to occur through downregulation of p21, a protein that degrades Beclin 1 (Portillo et al., 2010). Consistent with the evidence that the level of Beclin 1 expression is linked to autophagic activity (Liang et al., 1999), CD40-induced Beclin 1 upregulation facilitates autophagic killing of *T. gondii* triggered by CD40 (Portillo et al., 2010). These three events act in synchrony, likely optimizing the ability of CD40 to stimulate autophagy and induce toxoplasmacidal activity. Finally, CD40 also promotes autophagy by activating PKR (Ogolla et al., 2013), a serine-threonine kinase that stimulates autophagy (Talloczy et al., 2002, 2006). These events are relevant to *T. gondii* since autophagic targeting and/or killing of the parasite induced by CD40 is dependent on CaMKKβ, AMPK, TNF-α, JNK1/2, Beclin 1 upregulation, p21 downregulation, and PKR (Subauste et al., 2007; Portillo et al., 2010; Ogolla et al., 2013; Liu et al., 2016).

Animal studies support the importance of autophagy for control of *T. gondii* in the brain and eye. Autophagy-deficient *BECN1*^+/−^ mice, mice with deficiency of the autophagy protein ATG7 targeted to microglia/macrophages (Atg7^flox/flox^-Lyz-M Cre mice) and PKR^−/−^ mice are susceptible to cerebral and ocular toxoplasmosis (Portillo et al., 2010; Ogolla et al., 2013). This susceptibility is not explained by defects in cellular or humoral immunity against the parasite. Moreover, macrophages/microglia from these mice exhibit impaired killing of *T. gondii* in response to CD40 but not IFN-γ stimulation (Portillo et al., 2010; Ogolla et al., 2013).

### *T. gondii* Manipulates Host Cell Signaling to Avoid Targeting by Autophagy

Avoidance of the lysosomal compartment is essential for *T. gondii* survival. Autophagy is a constitutive process in eukaryotic cells. Moreover, a fraction of host cells is unable to exhibit autophagic targeting and VLF of intracellular tachyzoites despite activation through CD40. These findings suggest that *T. gondii* uses strategies to avoid targeting by autophagosomes. Indeed, the parasite activates host cell signaling pathways that achieve this purpose.

**Figure 2:**
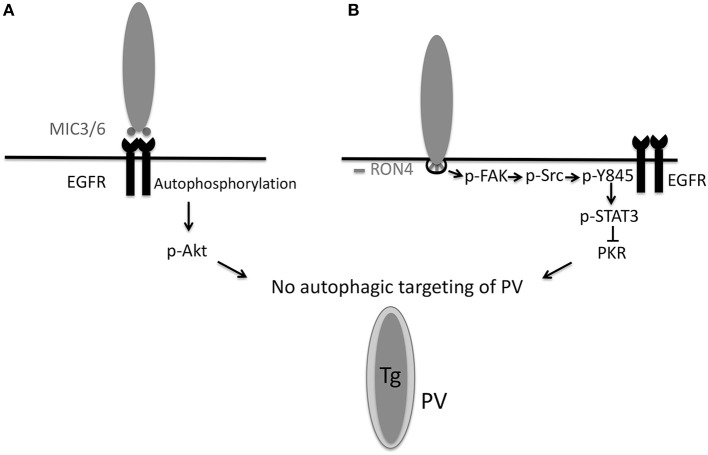
*T. gondii* invasion of host cells activates signaling cascades that prevent autophagic targeting of the parasite. **(A)** During attachment to host cells, *T. gondii* MIC3 and MIC6 cause EGFR autophosphorylation, leading to PI3K-dependent activation of Akt. Blockade of EGFR, PI3K, or Akt leads to autophagic targeting of the PV and killing of the parasite. **(B)** During invasion of host cells, the formation of the moving junction, characterized by expression of RON4, is accompanied by activation of FAK. In turn, FAK activates Src causing Src-dependent transactivation of EGFR (Y845 phosphorylation). This unique form of EGFR activation recruits STAT3 signaling that prevents activation of PKR and eIF2α. Blockade of the signaling cascade results in activation of PKR and eIF2α leading to autophagic targeting and killing of the parasite. Inhibition of the MIC3/6
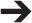
EGFR
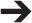
Akt or the FAK
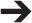
Src
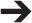
pY845 EGFR
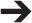
STAT3 pathways results in autophagic killing of *T. gondii* that is dependent on ULK1, Beclin 1, ATG7, and lysosomal enzymes. Modified with permission from Portillo et al. (2017) *PLoS Pathog* 13, e1006671.

Epidermal growth factor receptor (EGFR) is expressed in various cells various cell types (including epithelial cells, endothelial cells, microglia, and certain neurons) and can inhibit autophagy (Sobolewska et al., 2009). EGFR is composed of extracellular (ligand binding), transmembrane, intracellular tyrosine kinase and carboxyl-terminal tail domains (Purba et al., 2017). Ligand binding causes a conformational change in the kinase domain leading to activation of EGFR through autophosphorylation of tyrosine residues in the carboxyl-terminal tail (Purba et al., 2017). These phosphorylated residues recruit signaling molecules downstream of EGFR (Purba et al., 2017). *T. gondii* induces phosphorylation of the tyrosine residues 1,068, 1,148, and 1,173 of EGFR during infection of human or rodent cells (Muniz-Feliciano et al., 2013). *T. gondii*-induced EGFR signaling leads to activation of PI3K (Muniz-Feliciano et al., 2013), a molecule that triggers production of phosphatidylinositol 3,4,5 trisphosphate (PIP3). Transfection of host cells with a plasmid encoding GFP-tagged amino-terminal pleckstrin homology (PH) domain of Akt that binds PIP3 revealed PIP3 accumulation around the PV (Muniz-Feliciano et al., 2013). Consistent with the fact that PIP3 production is a major trigger of Akt activation, *T. gondii* induces Akt activation (Muniz-Feliciano et al., 2013) (Figure 2A). While parasite-induced Akt activation in macrophages is impaired by Pertussis Toxin (PTx) suggesting that Akt signaling can be dependent on G-protein coupled receptors (GPCR) (Kim and Denkers, 2006), genetic and pharmacologic blockade of EGFR in various cell types including macrophages/microglia revealed that EGFR is an important driver of Akt activation triggered by *T. gondii* (Muniz-Feliciano et al., 2013).

Inhibition of the EGF
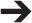
Akt pathway results in spontaneous recruitment of LC3 and formation of a double membrane structure around the PVM followed by VLF (Muniz-Feliciano et al., 2013). In both human and mouse cells, ensuing killing of type I and II strains of *T. gondii* is dependent on ULK1, Beclin 1, ATG7, and lysosomal enzymes (Muniz-Feliciano et al., 2013). These results are likely explained by the fact that Akt is a negative regulator of autophagy via activation of mTORC1 (Menon et al., 2014). Given that Akt activation is linked to inhibition of apoptosis of *T. gondii*-infected cells (Kim and Denkers, 2006), parasite-induced EGFR-Akt signaling may promote parasite survival by preserving the non-fusogenic nature of the PV and by avoiding death of infected cells subjected to pro-apoptotic signals.

EGFR can be activated by transmembrane proteins that are shed from the plasma membrane as a consequence of the ADAM (a disintegrin and metalloprotease) family of zinc-dependent metalloproteases (Yarden and Sliwkowski, 2001). This process is stimulated by GPCR (Yarden and Sliwkowski, 2001). However, treatment with GM6001, a broad-spectrum ADAM inhibitor, or with PTx fails to inhibit *T. gondii*-induced EGFR activation (Muniz-Feliciano et al., 2013). The parasite causes EGFR phosphorylation at tyrosine 1,148 (Muniz-Feliciano et al., 2013), a site that appears to be phosphorylated only by ligand binding to EGFR (Moro et al., 2002). In this regard, MIC3, MIC6, MIC8 have multiple domains with homology to EGF (Meissner et al., 2002). Recombinant MIC3 and MIC6 but not MIC4 or M2AP induce EGFR-Akt signaling in mammalian cells and impair the ability of CD154 to induce LC3 accumulation around the parasite (Muniz-Feliciano et al., 2013). In addition, MIC1 ko (deficient in MIC6), MIC3 ko and especially MIC1/3 ko parasites are defective in induction of EGFR-Akt activation (Muniz-Feliciano et al., 2013). While cells infected with MIC1/3 ko *T. gondii* do not exhibit spontaneous targeting by LC3^+^ structures, there is increased recruitment of LC3 and susceptibility to killing after incubation with stimulators of autophagy (CD154, Rapamycin) (Muniz-Feliciano et al., 2013). The likely explanation for these results is that MIC1/3 ko *T. gondii* have residual ability to induce EGFR-Akt signaling (Muniz-Feliciano et al., 2013). Although MIC8 has EGF-like domains, MIC8 ko parasites show no defect in EGFR activation (Muniz-Feliciano et al., 2013). These findings are likely explained by the fact that MIC8 ko parasites are not defective in host cell attachment and secrete MICs (Kessler et al., 2008). Whether simultaneous deficiency in MIC3, MIC6 and MIC8 ablates EGFR autophosphorylation or whether there is another mechanism that contributes to EGFR autophosphorylation remains to be determined. Taken together, in addition to being key for invasion of host cells, these studies indicate that MIC3 and MIC6 promote EGFR-Akt signaling to avoid lysosomal degradation of the parasite (Figure 2A).

Another mechanism operative in both human and murine cells that enables type I, II, and atypical strains of *T. gondii* to avoid targeting by autophagosomes is dependent on activation of Focal Adhesion Kinase (FAK), a cytoplasmic molecule that links extracellular signals to intracellular signaling cascades. *T. gondii* induces FAK activation at the level of the moving junction, an effect that is largely mediated by β integrins, presumably in the form of mechano-transduction-induced integrin clustering at the site of penetration of host cells (Portillo et al., 2017) (Figure 2B). Src becomes activated as a consequence of *T. gondii*-induced FAK activation (Portillo et al., 2017). Src can bind EGFR and transactivate this receptor even in the absence of ligand binding (Biscardi et al., 1999). EGFR transactivation is characterized by phosphorylation of a unique tyrosine 845 in the kinase domain of EGFR that recruits alternate signaling cascades downstream of EGFR including STAT3. Indeed, *T. gondii* triggers Src dependent phosphorylation of tyrosine 845 of EGFR followed by activation of STAT3 (Portillo et al., 2017) (Figure 2B), a negative regulator of autophagy (Van Grol et al., 2010; Shen et al., 2012). In the case of *T. gondii* infection, STAT3 activation prevents autophagic targeting of the parasite by impairing activation of the pro-autophagy protein PKR and its downstream signaling molecule eIF2α (Portillo et al., 2017). Genetic or pharmacologic blockade of any component of the FAK
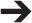
Src
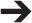
p845Y EGFR
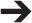
STAT3 pathway causes recruitment of LC3 around the parasite, VLF and parasite killing dependent on ULK1, Beclin 1, and lysosomal enzymes (Portillo et al., 2017). Thus, *T. gondii* activates an Akt- and a STAT3-dependent signaling pathway in both human and mouse cells to avoid autophagic targeting, and these pathways appear to function independently (Portillo et al., 2017) (Figure 2).

Animal studies have recently demonstrated the *in vivo* relevance of *T. gondii*-induced manipulation of host cell signaling in the pathogenesis of cerebral and ocular toxoplasmosis. The CNS is invaded via the blood stream when tachyzoites present in circulating infected leukocytes or extracellular tachyzoites reach the brain (Courret et al., 2006; Konradt et al., 2016). CNS invasion is preceded by infection of endothelial cells (Konradt et al., 2016). Expression of a dominant negative EGFR in endothelial cells ablates *T. gondii*-induced autophosphorylation and transactivation of EGFR (Lopez Corcino et al., 2019). Transgenic mice whose endothelial cells express DN EGFR exhibit diminished parasite load and histopathology in the brain and retina after *T. gondii* infection (Lopez Corcino et al., 2019). Mice with DN EGFR have reduced parasite load in these organs after i.v. administration of infected leukocytes or extracellular tachyzoites (Lopez Corcino et al., 2019). This protective effect is not explained by enhanced immunity or reduced leukocyte recruitment into the CNS. Rather, the effect of DN EGFR is to reduce the foci of infection in neural endothelial cells (Lopez Corcino et al., 2019). DN EGFR in these cells results in the spontaneous recruitment of LC3 around *T. gondii*, VLF and parasite killing dependent on ULK1 and lysosomal enzymes (Lopez Corcino et al., 2019). Moreover, *in vivo* administration of autophagy inhibitor 3-methyl adenine prevents DN EGFR mice from exhibiting reduced CNS invasion (Lopez Corcino et al., 2019). Altogether, EGFR is a novel regulator of *T. gondii* invasion of neural tissue, enhancing invasion likely by promoting survival of the parasite within endothelial cells through avoidance of autophagic targeting.

Although *T. gondii* activates signaling molecules that can inhibit autophagy, *T. gondii* does not prevent autophagosome formation in infected cells (Wang et al., 2009). In fact, *T. gondii* increases LC3-II levels and autophagosome formation in host cells at 24 h post-infection (Wang et al., 2009). These studies together with the demonstration that *T. gondii* induces lipophagy in host cells to obtain fatty acids (Pernas et al., 2018) would support that the parasite co-opts host cell autophagy to gain access to nutrients for its growth (Wang et al., 2009; Pernas et al., 2018). A model has been proposed whereby *T. gondii* utilizes the autophagy machinery of permissive (non-activated) host cells for its own benefit, whereas host cell autophagy would lead to parasite killing only in immune-activated host cells (Latre De Late et al., 2017). However, the signaling studies described above revealed an additional layer of complexity. They begin to indicate that CD40 and *T. gondii* have opposing effects on signaling molecules that regulate autophagy (i.e., PKR). The balance between these opposing effects may determine whether autophagic targeting of the parasite takes place. Host cell autophagy would cause parasite killing not only in CD40-activated host cells but also in resting cells if the effects of *T. gondii* on EGFR
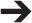
Akt or FAK/Src
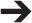
p-Y845 EGFR
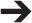
STAT3 signaling are blocked. This model has therapeutic implications since, for example, addition of EGFR tyrosine kinase inhibitors to resting cells restricts *T. gondii* growth (Muniz-Feliciano et al., 2013; Yang et al., 2014). Host cell autophagy would be beneficial to the parasite in resting host cells as long as the parasite is able to activate negative regulators that prevent autophagosomes from targeting and killing the parasite. In these cells, activation of the regulatory signaling cascades would not appear to inhibit global autophagy but rather would impair targeting of the parasite by autophagosomes.

## Autophagy-Independent Effects of Autophagy Proteins During *T. gondii* Infection

### IFN-γ Restricts *T. gondii* Through Autophagy-Independent Effects of Autophagy Proteins

IFN-γ is a major activator of effector responses against *T. gondii* in mammalian cells. IFN-γ causes vesiculation and rupture of the PVM in mouse cells leading to parasite release into the cytoplasm and parasite death (Martens et al., 2005; Zhao et al., 2008, 2009). While autophagosome-like structures can be noted around the parasites (Ling et al., 2006), the function of these structures is not to kill the parasite but likely to clear parasite/PVM fragments (Zhao et al., 2008; Choi et al., 2014; Ohshima et al., 2014). Indeed, parasite killing in IFN-γ-activated mouse cells is not mediated by lysosomal activity since expression of DN Rab7 that would inhibit lysosomal fusion and/or autolysosome maturation (Andrade et al., 2006) or incubation with lysosomal protease inhibitors fail to impair the anti-*T. gondii* activity induced by IFN-γ (Andrade et al., 2006; Van Grol et al., 2013; Choi et al., 2014). Thus, *bona fide* autophagy does not mediate the anti-*T. gondii* effects of IFN-γ.

**Figure 3:**
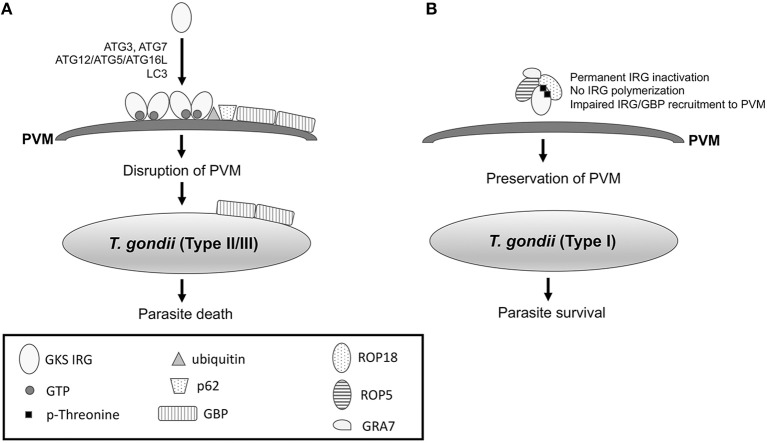
IFN-γ-induced recruitment of IRG and GBP to the PVM in mouse cells leads to killing of *T. gondii*, an effect that is prevented by virulent strains of the parasite. **(A)** IFN-γ induces expression of IRGs and GBPs in mouse cells. In cells infected with type II or III strains of *T. gondii*, IFN-γ causes recruitment of GKS IRGs to the PVM, an effect that is mediated by ATG3, ATG7, ATG12-ATG5-ATG16L, and LC3. IRGs drive ubiquitin deposition and p62-mediated recruitment of GBP to the PVM. IRGs and GBP disrupt PVM enabling GBPs to bind the surface membrane of the parasite leading to parasite death. **(B)** In mouse cells infected with type I *T. gondii*, ROP5/ROP18/GRA7 form a complex with IRG causing threonine phosphorylation. As a result, IRGs remain in an inactive GDP-bound conformation that prevents their oligomerization and loading into the PVM. ROP17 also phosphorylates threonine residues of IRG (see text). The models shown represent events that take place in mouse cells. Mechanisms operative in human cells are described in the text.

Interestingly, while autophagosomes are not involved in killing of susceptible strains of *T. gondii* within IFN-γ-activated cells (Zhao et al., 2008), selected autophagy proteins are required for parasite death in mouse cells. These autophagy proteins function by promoting recruitment of Immunity Regulated GTPases (IRGs) and Guanylate Binding Proteins (GBPs) to the PVM (Figure 3A). IFN-γ induces recruitment and loading of effector GKS subfamily of IRGs (Irga6, Irgb6, Irgb10, Irgd) onto the PVM causing its disruption (Martens et al., 2005; Ling et al., 2006; Khaminets et al., 2010). IRGs promote ubiquitin deposition on the PVM followed by p62-dependent recruitment of GBPs (Haldar et al., 2015) (Figure 3A). Disruption of the PVM enables GBPs to bind and kill the parasite (Kravets et al., 2016). ATG5 is required for recruitment of GKS IRGs (Irga6, Irgb6, Irgd) and mGBP1 to the PVM in IFN-γ-activated mouse macrophages and fibroblasts (Zhao et al., 2008; Khaminets et al., 2010; Selleck et al., 2013). Similarly, ATG7 and ATG16L1 are necessary for Irgb6 and mGBP1-5 recruitment (Choi et al., 2014; Ohshima et al., 2014) while ATG3 is required for recruitment of Irgb6, Irb10 and mGBP1-5 in fibroblasts (Choi et al., 2014; Haldar et al., 2014). Consistent with the fact that ATG3, ATG7, and the ATG12-ATG5-ATG16L1 complex mediate LC3 conjugation, LC3 is recruited to the PVM in IFN-γ-activated mouse macrophages and fibroblasts (Choi et al., 2014; Park et al., 2016). The LC3 homologs gamma-aminobutyric acid-A-receptor-associated proteins (GABARAPs) are also recruited to the PVM in a conjugation-dependent manner (Park et al., 2016). These proteins target IRGs to the PVM in mouse cells (Park et al., 2016). In another study, GABARAPL2 (Gate-16) but not LC3 was required for recruitment of Irga6 and GBP1-5 to the vacuole of IFN-γ-treated mouse fibroblasts (Sasai et al., 2017).

As stated above, ATG proteins do not function through *bona fide* autophagy to restrict *T. gondii* in IFN-γ activated cells. Indeed, lysosomal degradation does not mediate the effects of these proteins (Choi et al., 2014). Moreover, ULK1 and ATG14L are not required in order for IFN-γ to control *T. gondii* (Choi et al., 2014). Similarly, ATG9, ATG14L and FIP200 are not required for recruitment of LC3, Irg6a and GBP to the vacuole (Choi et al., 2014; Ohshima et al., 2014; Sasai et al., 2017). While the mechanism of action of ATG proteins remains to be fully elucidated, these proteins may activate IRGs (Haldar et al., 2014), LC3 may target IRGs to the membrane (Park et al., 2016), and Gate-16 associates with the small GTPase ADP-ribosylation factor 1 (Arf1) to mediate IRG recruitment (Sasai et al., 2017).

IRGs represent the major mechanism by which IFN-γ protects mice during the acute phase of *T. gondii* infection (Martens et al., 2005). Consequently, ATG proteins not only mediate the anti-*T. gondii* activity in mouse cells activated by IFN-γ but they are also required for *in vivo* protection. Mice with deficiency in ATG5, ATG7, or ATG16L targeted to phagocytes exhibit marked susceptibility to acute infection with *T. gondii* (Zhao et al., 2008; Choi et al., 2014). In contrast, ATG14L deficiency does not increase susceptibility to acute infection (Choi et al., 2014). In addition, Gate-16^−/−^ mice succumb to acute infection with *T. gondii* in a manner that mimics IFN-γ^−/−^ mice (Sasai et al., 2017).

The effector mechanisms activated by IFN-γ to restrict *T. gondii* growth in human cells are less well-characterized and differ from those in mouse cells. Mechanisms in human cells appear to be cell-type specific and are reported to include induction of indoleamine 2,3-dioxygenase that deprives the parasite from tryptophan (Pfefferkorn, 1984) and host cell death that results in early parasite egress without replication (Niedelman et al., 2013). In contrast to mice, humans express only 2 IRGs that cannot be induced by IFN-γ explaining why IRGs do not mediate the effects of IFN-γ in human cells (Bekpen et al., 2005). Human cells express GBPs (Ohshima et al., 2014). Moreover, hGBP1-5 are recruited to the parasite in an ATG16L-dependent manner in IFN-γ-activated human HAP1 cells (Ohshima et al., 2014). However, GBPs are not required for restriction of *T. gondii* (Ohshima et al., 2014). Studies in human epithelial (HeLa) cells identified a mechanism for control of type II and type III *T. gondii* induced by IFN-γ that is dependent on ubiquitination and some ATG proteins (Selleck et al., 2015). IFN-γ induces ubiquitination of the PV and recruitment of the ubiquitin adaptor proteins p62 and Nuclear Domain 10 Protein 52 (NDP52), as well as LC3 (Selleck et al., 2015). These vacuoles become surrounded by multiple layers of host membrane that would restrict parasite growth (Selleck et al., 2015). While this process is dependent on ATG16L and ATG7, it occurs independently of Beclin 1 and does not cause VLF, indicating that it does not represent autophagy (Selleck et al., 2015).

IFN-γ was reported to restrict *T. gondii* growth within human endothelial cells through a mechanism that remained to be identified (Woodman et al., 1991). A recent study revealed that vacuoles containing type II *T. gondii* within human endothelial cells are targeted by K63-linked ubiquitin in response to IFN-γ (Clough et al., 2016). This is followed by recruitment of p62 and NDP52, acidification of the vacuole and parasite death. This process does not represent autophagy since it is not accompanied by recruitment of ATG16L, GABARAP, and LC3 (Clough et al., 2016). Moreover, in contrast to IFN-γ-activated HeLa cells, the ability of IFN-γ to restrict parasite growth in human endothelial cells is not dependent on ATG16L (Clough et al., 2016). Taken together, there are two novel cell-type specific mechanisms by which IFN-γ restricts the parasite growth in human non-hematopoietic cells. These mechanisms involve ubiquitination of the vacuole followed by either the formation of a multilayer structure around the vacuole or vacuole acidification.

### *T. gondii* Impairs Recruitment of IRGs to the PVM

The ability of IRGs to protect mice against *T. gondii* depends on the parasite strain. While virulent type I *T. gondii* prevents recruitment of IRGs to the PVM and avoids eradication, low virulence type II strains and avirulent type III strains of *T. gondii* cannot avoid IRG recruitment and are thus eradicated (Zhao et al., 2009; Khaminets et al., 2010). Evasion of IRG recruitment is mediated by parasite proteins released within host cells during invasion. The rhoptry protein ROP18 is a polymorphic protein kinase and a major determinant of parasite virulence in mice (Saeij et al., 2006; Taylor et al., 2006). Type I *T. gondii* secretes a catalytically active form of ROP18 that phosphorylates IRGs at two threonine residues in the nucleotide-binding domain (Fentress et al., 2010; Steinfeldt et al., 2010) (Figure 3B). As a result, the GTPase function of IRGs is inhibited and their oligomerization and loading into the PVM are impaired (Fentress et al., 2010; Steinfeldt et al., 2010). The ability of ROP18 to phosphorylate IRGs is dependent on the presence of virulent alleles of *ROP5*. ROP5 are a group of catalytically inactive kinases (pseudokinases) that control parasite virulence in mice (Behnke et al., 2011; Reese et al., 2011). ROP5 proteins bind a conserved surface of IRG and promote that IRG remain in an inactive GDP-bound conformation (Fleckenstein et al., 2012; Reese et al., 2014). As a result, GTP-dependent activation of IRG is prevented, and threonines in the nucleotide-binding domain become exposed, enabling their phosphorylation by ROP18 and permanent inactivation of IRG (Fleckenstein et al., 2012; Reese et al., 2014). Thus, ROP5 appears to act as an allosteric regulator of ROP18 (Reese et al., 2014) and is required for the catalytic activity of ROP18 (Behnke et al., 2012). Indeed, virulent forms of both ROP5 and ROP18 are required to prevent IRG recruitment to the PVM. ROP18 and ROP5 largely explain the differences in virulence in mice among type I, II, and III strains (Saeij et al., 2006; Taylor et al., 2006). Despite encoding *ROP18* that is likely catalytically active, type II strains cannot prevent IRG recruitment because they carry alleles of *ROP5* that do not assist IRG phosphorylation by ROP18. In addition, type III strains carry a high-virulence allele of *ROP5* but are avirulent because of their minimal expression of *ROP18*. The allelic combination of *ROP18* and *ROP5* genes also determines the virulence of atypical strains of *T. gondii* (Niedelman et al., 2012).

In addition to ROP18 and ROP5, ROP17 also contributes to *T. gondii* virulence in mice (Etheridge et al., 2014). ROP17 associates with ROP5 and phosphorylates threonine residues of IRG (Etheridge et al., 2014). However, in contrast to ROP18, the *in vitro* activity of ROP17 does not require ROP5 (Etheridge et al., 2014). Finally, the dense granule protein GRA7 is another component of the ROP18-ROP5 complex and modulates IRG recruitment to the PVM (Hermanns et al., 2016) (Figure 3B). GRA7 appears to associate with ROP5 and functions by allowing efficient ROP18 kinase activity (Hermanns et al., 2016).

In summary, important advances have been achieved in our understanding of how autophagy proteins and autophagy attack *T. gondii*-containing vacuoles within host cells. Given that maintaining the integrity of this niche is essential to parasite survival, it is not surprising that *T. gondii* utilizes various strategies to counteract the effects of autophagy proteins and autophagy. Pharmacologic approaches to enhance autophagy for therapeutic purposes may be complicated by the homeostatic role of autophagy in various cellular processes, the complexity of autophagy cascades, and the specificity of pharmacologic agents. Strategies to prevent *T. gondii* from blocking autophagic targeting may represent a more feasible avenue to develop novel ancillary approaches to improve the treatment of toxoplasmosis.

## Author Contributions

The author confirms being the sole contributor of this work and has approved it for publication.

### Conflict of Interest Statement

The author declares that the research was conducted in the absence of any commercial or financial relationships that could be construed as a potential conflict of interest.
